# Fluid accumulation syndrome in sepsis and septic shock: pathophysiology, relevance and treatment—a comprehensive review

**DOI:** 10.1186/s13613-024-01336-9

**Published:** 2024-07-20

**Authors:** Carmen Andrea Pfortmueller, Wojciech Dabrowski, Rob Wise, Niels van Regenmortel, Manu L. N. G. Malbrain

**Affiliations:** 1 Department of Intensive Care, Inselspital, Bern University Hospital and University of Bern, Freiburgstrasse 10, 3010 Bern, Switzerland; 2 First Department of Anaesthesiology and Intensive Therapy, Medical University of Lublin, Lublin, Poland; 3 Department of Anaesthesia and Critical Care, School of Clinical Medicine, University of KwaZulu-Natal, Durban, South Africa; 4 Faculty Medicine and Pharmacy, Vrije Universiteit Brussel (VUB), Brussels, Belgium; 5 Intensive Care Department, John Radcliffe Hospital, Oxford University Trust Hospitals, Oxford, UK; 6 Department of Intensive Care Medicine, Ziekenhuis Netwerk Antwerpen Campus Stuivenberg/Cadix, Antwerp, Belgium; 7 Department of Intensive Care Medicine, Antwerp University Hospital, Antwerp, Belgium; 8 International Fluid Academy, Lovenjoel, Belgium; 9 Medical Data Management, Medaman, Geel, Belgium

**Keywords:** Fluids, Resuscitation, De-resuscitation, Fluid accumulation, Safety, Monitoring

## Abstract

In this review, we aimed to comprehensively summarize current literature on pathophysiology, relevance, diagnosis and treatment of fluid accumulation in patients with sepsis/septic shock. Fluid accumulation syndrome (FAS) is defined as fluid accumulation (any degree, expressed as percentage from baseline body weight) with new onset organ-failure. Over the years, many studies have described the negative impact of FAS on clinically relevant outcomes. While the relationship between FAS and ICU outcomes is well described, uncertainty exists regarding its diagnosis, monitoring and treatment. A stepwise approach is suggested to prevent and treat FAS in patients with septic shock, including minimizing fluid intake (e.g., by limiting intravenous fluid administration and employing de-escalation whenever possible), limiting sodium and chloride administration, and maximizing fluid output (e.g., with diuretics, or renal replacement therapy). Current literature implies the need for a multi-tier, multi-modal approach to de-resuscitation, combining a restrictive fluid management regime with a standardized early active de-resuscitation, maintenance fluid reduction (avoiding fluid creep) and potentially using physical measures such as compression stockings.

*Trial registration*: Not applicable.

**Graphical Abstract:**

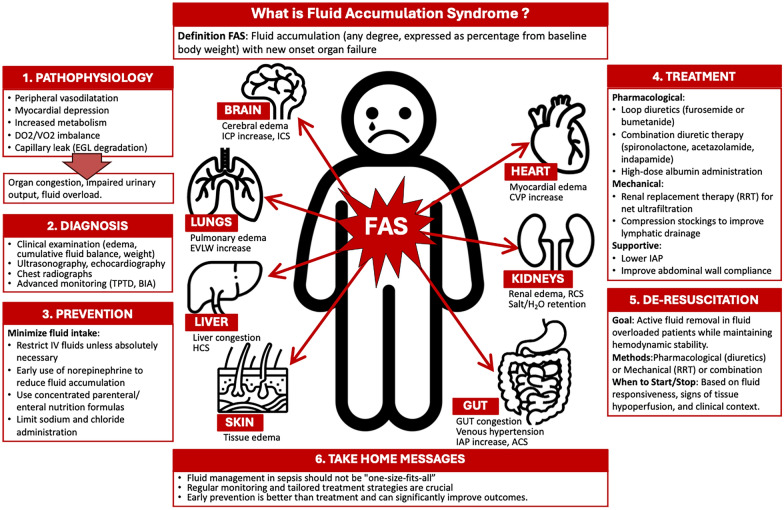

## Introduction

Fluids are widely used in critically ill patients to restore hemodynamic stability and tissue perfusion [1]. Fluid accumulation (FA) is very common in critical illness and occurs in at least 20% of the ICU population, particularly in patients with increased capillary leak due sepsis and septic shock [2, 3]. Many studies have previously described the negative impact of fluid accumulation syndrome (FAS) on clinically relevant outcomes. Several observational trials [4–9], as well as one meta-analysis (of mainly observational trials) [10], suggest that FAS is associated with increased mortality in critically ill patients. However, current RCTs on the topic have not found a mortality benefit with restrictive fluid management [11, 12] and protocolized de-resuscitation [13]. While FAS is a well described entity in critical care, with a serious impact on patient outcomes, its monitoring, prevention and treatment is less well described, with much uncertainty. In this review, we aimed to comprehensively summarize current literature on pathophysiology, relevance, diagnosis and treatment of fluid accumulation in patients with sepsis/septic shock in line with the ROSE framework (with the resuscitation—optimization—stabilization and evacuation phases) [14, 15].

## What is FAS?

**Fig. 1:**
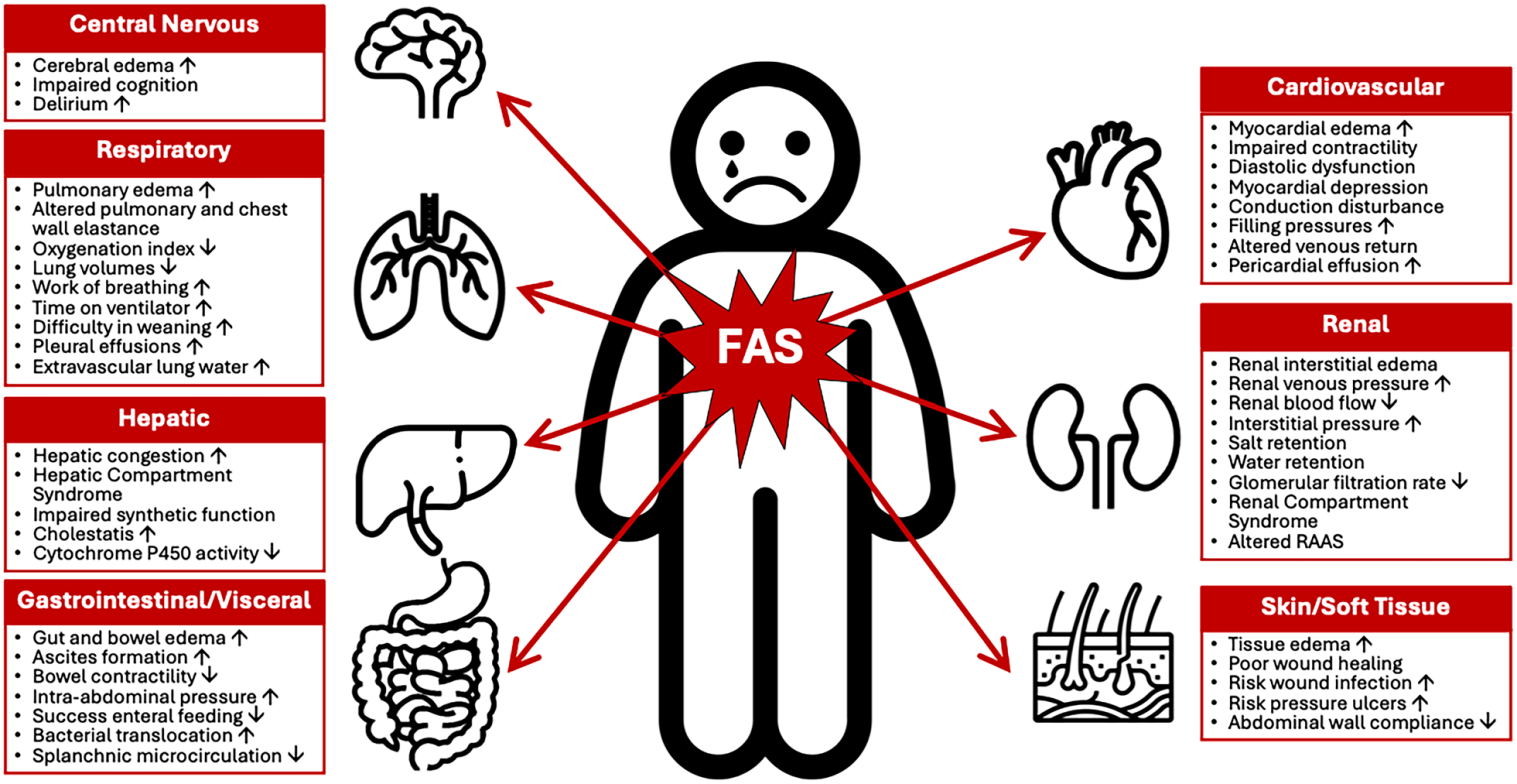
Potential adverse consequences of fluid accumulation. Adapted with permission from Malbrain et al. according to the Open Access CC BY License 4.0 [15–17]. Effects mentioned are related to the setting of sepsis, capillary leak and fluid accumulation. I.e. the numbness refers to the presence of peripheral edema and anasarca that may cause skin conduction disturbances, compression of nerves, reduced blood flow and reduced mobility. Additionally, severe and prolonged fluid imbalances can lead to a range of health issues and complications, including electrolyte imbalances, which may indirectly affect the body's ability to respond to stress, including the production of cortisol by the adrenal glands. *APP*: abdominal perfusion pressure (MAP minus IAP), *RSB*: rapid shallow breathing, *HCS*: hepatic congestion, *GRV*: gastro-esophageal reflux, CARS: cardiac-renal syndrome, *AKI*: acute kidney injury, JVP: jugular venous pressure, *HJR*: hepato-jugular reflux

Fluid accumulation may be defined and calculated by dividing the cumulative fluid balance by the baseline body weight. Please see testbox 1 for a critical appraisal of this definition. FAS is defined as any degree of fluid accumulation (expressed as a percentage) with new onset organ failure (which may be described by a sequential organ failure assessment (SOFA) organ sub-score equal to or greater than 3) that may be due to FA, [16]. Most organ systems, including the lungs, heart, and gastrointestinal tract, are negatively affected by FAS (see Fig. 1 for an overview) [16].

**Textbox 1:**
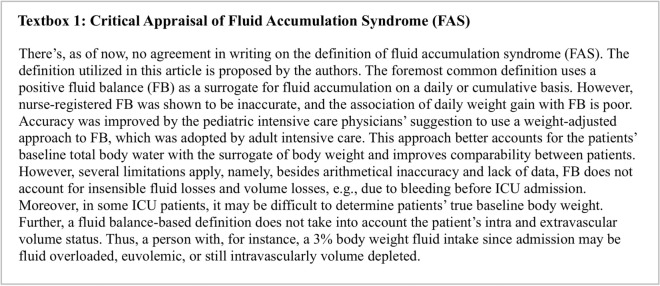
Critical appraisal of FAS definition. *FB*: fluid balance

In sepsis and septic shock, a cascade of circulatory effects, such as peripheral vasodilatation, myocardial depression, and increased metabolism, lead to an imbalance between systemic oxygen delivery and oxygen demand, resulting in global tissue hypoxia or shock [18, 19]. Sepsis and septic shock are not always associated with a volume depleted state, but rather it is the microcirculatory alterations together with vasodilatation and potentially cardiac dysfunction (myocardial depression) that lead to a reduction in stressed volume and cardiac output [20]. Stressed volume refers to the portion of circulating volume that actively contributes to tissue perfusion by being in direct contact with the vessel walls and thus exerting pressure against the walls [21–23]. Thus, the idea of fluid resuscitation in patients with sepsis is to increase the stressed volume and mean systemic filling pressure (P_msf_), thereby increasing cardiac preload via an increased gradient for venous return [the difference between P_msf_ and central venous pressure (CVP)] [20]. In an international observational study it was shown that on average around fifty percent of ICU patients showed a response to a fluid bolus [24]. Importantly, while the ANDROMEDA-Shock study revealed that initially more than 50% of their study population was fluid responsive, this proportion decreased substantially during the intervention period [25, 26]. In clinical practise however, a large multi-centre cohort study demonstrated that a significant percentage of actually fluid unresponsive patients receive IV fluids on the ICU (approximately 50%) [24].

A profound inflammatory state, such as sepsis and septic shock, results in the activation of inflammatory mediators that initiate and perpetuate the degradation of endothelial glycocalyx, causing capillary leak [27]. The latter also decreases colloid oncotic pressure and impacts fluid haemostasis. Recent data suggest that IV fluid administration may promote this effect [27], leading to a vicious cycle through amplification of endothelial dysfunction.

A further factor warranting consideration is organ congestion. The clinical impact of “venous congestion” in sepsis/septic shock is well described in the kidneys [28–30]. In a physiological state, both kidneys receive roughly a quarter of the cardiac output, whereas in a shocked state, such as the first phase of sepsis/septic shock (R and O of the ROSE model), this can decrease to 10% or less in order to divert blood flow away from the renal bed [31–33]. Urinary output/kidney function may decline as a result. However, in the stabilization and de-resuscitation phases of sepsis/septic shock, (phases S and E of the ROSE model) urinary output may still remain low despite the initial shock state having improved. However, in these stages, “renal venous congestion”, may cause impaired urinary output and kidney function i.e., because of FAS, intra-abdominal hypertension, abdominal compartment syndrome and/or right heart failure. This impaired renal venous outflow triggered kidney injury is associated with mortality in the critically ill [28, 29]. Unfortunately, the still low urinary output/impaired kidney function may (wrongly) entice physicians to administer additional IV fluid with the aim of increasing urinary output, creating a vicious cycle.

## How do I recognize and monitor FAS?

There are many modalities to diagnose and monitor FAS, but there is currently no gold standard [34]. Clinical examination may provide valuable clues for detection of fluid accumulation, such as peripheral (pitting or anasarc) edema, respiratory distress and (prolonged) circulatory failure without clear lung/cardiac pathology. These are rather non-specific and may not reflect intravascular fluid status and are hard to distinguish from other causes of organ failure. Focussed ultrasonography and echocardiography may provide further insights whether organ failure might be related to fluid accumulation (i.e., VExUS score, increased end-diastolic volumes, inferior vena cava collapsibility index). Chest radiographs have been one of the most commonly used (albeit non-specific) tests to evaluate hypervolemia. Radiographic signs of volume overload include dilated upper lobe vessels, cardiomegaly, interstitial edema, enlarged pulmonary artery, pleural effusions, alveolar edema, prominent superior vena cava, and Kerley B-lines [35]. Bedside ultrasonography can examine all these signs and is a useful diagnostic tool for assessing pulmonary congestion [35, 36]. Advanced cardiac monitoring tools that may be of use include transpulmonary thermodilution [e.g., with PICCO (Getinge, Sölna, Sweden) or Volumeview (Edwards Lifesciences, Irvine, California, USA)] that provides information on extravascular lung water and pulmonary vascular permeability index. Furthermore, bioelectrical impedance analysis (BIA) is a less well known, but fully non-invasive, and inexpensive monitor [37]. BIA is a conductive property-based method of detecting soft tissue hydration with a 2–3% measurement error [37–39]. It is considered an easy and sensitive method to assess total body water (TBW), extracellular water content (ECW), intracellular water content (ICW), the ECW/ICW ratio overhydration (OH), volume excess, body cell mass (BCM) and derived phase angle [40–43].. Further validation of this tool in clinical practice and specific patient populations is needed but initial studies in ICU patients show promising results [43, 44]. Depending on the clinical context and resources available, the authors suggest a combination of non-invasive parameters like cumulative fluid balance, clinical assessment, BIA-derived parameters and ultra-sound/echocardiography for initial assessment and monitoring of FA.

## How can FAS be prevented?

Prevention of FAS is probably the best treatment, please see Table 1 for an overview on prophylactic measures. Currently proposed strategies are de-escalation/ minimization, fluid restriction and small fluid resuscitation by means of albumin [45].

**Table 1:** Monitoring and prevention of fluid accumulation syndrome (FAS)

NOTE: Fluids should only be administrated when hypovolemia, fluid responsiveness AND signs of impaired tissue perfusion are present	
**Monitoring**	
– Basic monitoring (i.e. arterial and central venous line),	
– In case of unresolved shock consider echocardiography and advanced hemodynamic monitoring (eg. pulse contour or transpulmonary thermodilution)	
– Obtain baseline body weight (scale, estimate, retrieve from medical records)	
– Monitor for risk for fluid accumulation: i.e. daily body weight, daily and cumulative fluid balance, edema formation, sonography	
– Assess for impaired end-organ function: IAP, APP, PF ratio, success of EN, EVLWI, PVPI, BIA	
– Assess fluid responsiveness with functional hemodynamics (i.e. PPV or SVV, passive leg raising test, end-expiratory occlusion test)	
– Assess for signs of tissue hypoperfusion (DO_2_/VO_2_ mismatch; i.e. elevated lactate, increased mottling score, increased capillary refill time)	
**Prevention**	
**A) Fluids are required:**	**B) Fluids are not required: de-escalation**
– Use a restrictive fluid management regime	– Limit fluid intake
– When maintenance fluids are necessary, opt for balanced and sodium-poor alternatives (NaCl 0.18–0.45%)	– Fluid withdrawal lower volume and speed)
– Use low-chloride alternatives to NaCl 0.9% when selecting resuscitation and replacement fluids	– Limit sodium intake
– Frequently re-assess preload and fluid responsiveness/tissue perfusion, only administer fluids in fluid responsive patients	– Limit/avoid maintenance solutions
– Consider early norepinephrine use	– Limit/avoid fluid creep
– Stop fluid administration once fluid responsiveness and/or tissue perfusion are absent	– Improve lymphatic drainage (i.e. use leg compression bandages)
– Consider the (early) use of albumin 20%, especially when serum albumin levels are low (< 30 g/L) [46]	– Use high density or concentrated enteral formula’s (i.e. 2 kcal/mL)

Table adapted with permission from Malbrain et al. according to the Open Access CC BY License 4.0 (ESM file) [16]. This table presents some suggestions for prevention of fluid accumulation based on personal experience of the co-authors. It does not aim to provide an exhaustive, graded and concise overview of the literature as current evidence is mostly limited to observational, retrospective or small clinical studies, and more randomized trials are needed to better establish a personalized approach to fluid management. For more information we refer the reader to some recent review papers on this topic [15, 47]

*EN*: enteral nutrition, *EVLWI*: extra-vascular lung water index, *FA*: fluid accumulation, *APP*: abdominal perfusion pressure (MAP minus IAP), *IAP*: intra-abdominal pressure, *PEEP*: positive end-expiratory pressure, *PF*: P_a_O_2_ over F_i_O_2_ ratio, *PPV*: pulse pressure variation, *SVV*: stroke volume variation, *BIA*: bio-electrical impedance analysis

De-escalation/minimization implies limiting fluid intake to avoid unnecessary intravenous fluid administration i.e. by only administrating IV fluids to patients who are hypovolemic, fluid responsive and show signs of shock with tissue hypoxia. Additionally, early administration of norepinephrine was shown to have a positive effect on cumulative fluid balance in a propensity-score matched analysis in 337 patients where patients were allocated either to the very early vasopressor group (< 1 h) or a delayed vasopressor group [48]. A recent study found that a bolus of fluid of the same volume has a greater hemodynamic effect and increase in mean systemic filling pressure at a high dose than at a low dose of norepinephrine during septic shock confirming a synergistic effect [49]. However, further research is needed.

Further measures are i.e., to switch medication from intravenous to oral (or nasogastric) where possible [14, 15] to minimize fluid creep (fluids given with medication and flushes), to use concentrated parenteral or enteral nutrition formulas, and to only give maintenance fluids that are required (see Table 2 for a definition of fluid types). Fluid creep and maintenance fluids are substantial contributors to overall fluid balance (> 60%), and thus a reduction in creep/maintenance fluid may considerably reduce total fluid input [50]. Fluid creep and maintenance fluids impose also a substantial sodium (and chloride) burden and may thereby perpetuate fluid retention [50]. Choosing a low salt maintenance fluid strategy resulted in 0.6 L less fluid accumulation in healthy volunteers within 48 h, and almost 1 L less in peri-operative patients [51–53]. Another important potential intervention is the use of more concentrated, high density nutritional formulations (2 kcal/mL). Nutrition accounts, on average, for approximately 25–33% of the total fluid intake in critically ill patients [50]. As current guidelines propose some form of enteral feeding (e.g., trophic) as early as possible [54], fluids administered with nutrition are a significant contributor to overall fluid balance. Thus, changing to a more concentrated enteral formula would allow for a substantial reduction in overall fluid intake [55]. These concepts warrant evaluation in high quality studies.

**Table 2:** Terminology

Resuscitation fluids	Resuscitation fluids refer to the fluids administrated in the early initial phase of shock to restore of adequate organ perfusion. They should only be given in case of shock (DO_2_/VO_2_ imbalance with increased lactate) AND low preload AND fluid responsiveness and they should always be given as a fluid challenge i.e. assessing fluid status and fluid responsiveness before and after. Most often they are given as a bolus of 4 mL/kg over 10–15 min [56]
Fluid Creep	A term that refers to the unintentional and unmeasured fluid volumes administered in the process of delivering other medication (antibiotics, sedatives, painkillers, etc.) and/or nutrition through enteral and parenteral routes
Maintenance fluids	Maintenance fluids are a source of water, electrolytes and also potentially glucose. The aim of maintenance fluid is to cover the daily needs and prevent dehydration and electrolyte disorders, if the patients need is not met through other sources (i.e. nutrition)
De-escalation	Refers to not initiating extra fluids (withhold) or lowering of the dose or speed of administration (withdraw/reduction) of previously started fluid therapy due to improvement in the clinical condition of the patient
De-resuscitation	Correction of fluid accumulation or fluid overload by active removal of the excess fluids using non-pharmacological mechanical (e.g., dialysis with net ultrafiltration) or pharmacological (e.g., diuretics) methods

Fluid creep may sum up to 33% of all fluids administered, compared to maintenance/replacement (25%), nutrition (33%) and resuscitation (6%) [50]

Another strategy proposed to prevent FAS is fluid restriction. Two large RCTs (CLASSIC, CLOVERS) failed to prove that restrictive fluid management regimens are superior to usual care in terms of mortality [12, 57]. The failure to change clinical outcomes may be explained by the substantial volume of fluids administered to patients before randomization (i.e., in the emergency or operating room) and the lack of minimizing fluid creep in the trial. Furthermore, as many pragmatic trials, the CLASSIC trial was not able to demonstrate a clear separation of total fluid volumes administered between the restrictive versus liberal groups. In addition, what has been defined as “restrictive” in one trial may have been “liberal” in another due to the lack of a current gold standard definition. In a recent meta-analysis of 13 RCTs, including almost 4000 patients, adverse events were similar in the liberal and restrictive fluid management groups [58]. However, the number of patients harmed by fluid restriction was similar to the number of patients it helped; potential benefit or harm cannot be excluded thus [58]. Similar results were found in a Bayesian analysis of the CLASSIC trial data [59].

Another measure to prevent FAS may lie in the administration of 20% albumin, which was shown to increase intravascular volume twofold [60]. In the clinical setting, the SWIPE, ALBIOS and even the SAFE trials demonstrated better fluid balances with the use of albumin [61–63], but without improving mortality [63].

## How can we treat FAS?

De-resuscitation specifically refers to late goal-directed fluid removal together with a late conservative fluid management strategy (see Table 2 for definitions), that involves active fluid removal using diuretics and renal replacement therapy (RRT) with net ultrafiltration [45]. The goal in treating FAS should be to increase diuresis and/or fluid removal, preferably following a multi-modal, multi-tier approach, as illustrated in Table 3

**Table 3:** Treatment of fluid accumulation syndrome (FAS) : De-resuscitation

NOTE: Fluids should only be removed when there is fluid UNresponsiveness AND absence of signs of impaired tissue perfusion AND presence of FAS (any percentage of fluid accumulation AND impaired end-organ function) and venous congestion. Low doses of norepinephrine are not a contraindication for de-resuscitation.
**A) Pharmacological measures**
– Perform furosemide stress test to assess tubular cell integrity (diagnostic test): patient passes test if UO > 200 ml over the next 2 hours after furosemide bolus of 1 mg/kg (naive) or 1.5 mg/kg (previous use)
– Apply loop diuretic (i.e. furosemide or bumetanide): high dose and continuous furosemide (1 mg/kg bolus and 10 mg/hr)
If de-resuscitation insufficient a combination therapy of diuretics may be applied:
– Carbonic Anhydrase Inhibitors (Acetazolamide, 250 – 500 mg IV bolus): inhibition of Na reabsorption in proximal tubule in case of metabolic alkalosis (BE > 5 mEq/L)
– Thiazide (Indapamide, 2.5 – 5 mg PO): inhibition of Na reabsorption in distal tubule in case of hypernatremia
– Potassium sparing (Spironolactone, 25 – 50 mg PO): aldosterone receptor antagonist, reduction of Na reabsorption at the collector duct (ENaC channel)

**B) Mechanical measures**
– Start RRT (continuous or intermittent) with net ultrafiltration to obtain a daily negative fluid balance of 12 to 24 ml/kg.*
**C) Supportive measures**
– Lower IAP and increase abdominal perfusion pressure (APP = MAP – IAP)
o improve abdominal wall compliance (sedation, neuromuscular blockers, body positioning)
o reduce intraluminal volume (ileus)
o reduce intra-abdominal volume (ascites)
– Increase/support cardiac function
o inotropes (i.e. dobutamine, milrinone)
o low dose vasopressors to maintain APP > 60 mmHg
– Use vasodilators (i.e. calcium antagonists)
o increase renal blood flow
– Apply lung protective ventilation with application of PEEP (to counteract IAP) and limiting driving pressures below 14 cmH_2_O

Table adapted with permission from Malbrain et al. according to the Open Access CC BY License 4.0 [16]. This table presents some suggestions for prevention of fluid accumulation based on personal experience of the co-authors. It does not aim to provide an exhaustive, graded and concise overview of the literature as current evidence is mostly limited to observational, retrospective or small clinical studies, and more randomized trials are needed to better establish a personalized approach to fluid management. For more information we refer the reader to some recent review papers on this topic [15, 47]

*APP*: abdominal perfusion pressure (MAP minus IAP), *BE*: base excess, *IAP*: intraabdominal pressure, *FAS*: fluid accumulation syndrome, *MAP*: mean arterial pressure, *PEEP*: positive end-expiratory pressure, *PO*: per os (orally), *RRT*: renal replacement therapy, *SOFA*: sepsis and organ failure assessment, *UO*: urine output, *VExUS*: venous congestion excess by ultrasound

*Target net ultrafiltration needs to be tailored to the individual patient and adjusted based on the haemodynamics and fluid requirements of the patient. It may need to be slower in patients with cardiogenic shock and higher in patients who are very fluid overloaded and also have large fluid requirements, i.e. blood products, total parenteral nutrition (TPN) etc

There is currently no established de-escalation or de-resuscitation strategy in the critically ill literature, and high quality RCTs are scarce. Several studies have demonstrated that a more progressive use of loop diuretics to achieve a greater volume of fluid removal in fluid overloaded patients (with or without AKI) is associated with improved outcomes [5, 64–66]. This is also true for critically ill patients that are still on vasopressor support [67]. Hence, the authors suggest that therapy with furosemide may be started independent of actual vasopressor doses once the criteria for de-resuscitation are met (i.e., the patient has FAS with venous congestion, is hemodynamically stable, fluid unresponsive, and shows no signs of tissue hypoperfusion). The dosing regimen for diuretic therapy should be based on pharmacodynamics and pharmacokinetic considerations, whereas the dose is dependent on the patient’s kidney function, previous exposure to the drug, and potential tolerance [68]. Doses may be titrated to achieve an output that is greater than the input [69]. If the response to furosemide therapy is limited, evidence from the heart failure population shows a combination therapy of diuretics may be considered using spironolactone, acetazolamide, or indapamide but this needs to be studied in mixed ICU poulations (Table 3) [70, 71]. Other studies showed the beneficial effect on fluid removal by using hyperoncotic 20% albumin preceding furosemide use [72], or the combination of PEEP levels set to counteract IAP, followed by 20% albumin and furosemide (PAL treatment) [45]. While there is conflicting data on the effect of PEEP levels on pulmonary edema. On the one hand, alveolar recruitment induced by PEEP may have effects on alveolar vessels. On the other hand, high PEEP may also be responsible of an increase in CVP, which represent the downstream pressure of the lymphatic drainage and may promote fluid accumulation. The PAL treatment as described herein was used in patients with increased IAP and the PEEP (in cmH_2_O) was set at the level of IAP (in mmHg) to counterbalance and neutralise the effects at the level of the diaphragm. This strategy also takes in to account the fact that on average the pressure transmission from the abdomen to thorax compartment is about 35–50%, since the conversion factor between cmH_2_O and mmHg is 1.36 (or thus 36%) [73]. However, the clinician should be aware of the interplay between IAP, PEEP and lymphatic drainage as well as heart–lung-abdomen interactions. The presence of mechanical ventilation with high PEEP reduces the lymph drainage further which together with the increase in IAP decreases the lymphatic pressure gradient in the splanchnic regions, thereby promoting fluid accumulation [74–76].

If a patient is already on RRT, mechanical fluid removal should be started if the patient meets the criteria for FAS. The total amount of fluid to be removed should be calculated based on the cumulative fluid balance and the current hemodynamic response. Once the criteria for de-resuscitation are met, active fluid removal by RRT should start, independent of the patient’s current vasopressor dose, as hypotension itself is not a criterion for hypovolaemia [67, 77]. Alternatively, intermittent haemodialysis may be used. Fluid removal is titrated to achieve a daily output that is greater than the input.

Transcapillary refill rate or plasma refill rate, which depends on the distribution of excess fluids (intravascular vs extravascular), should be considered for “dosing” of active de-resuscitation by means of diuretic therapy or RRT [78]. Loop diuretics mainly reduce circulating blood volume and thus reduce intravascular fluid overload. In a delayed fashion (triggered by osmotic shifts), fluids then translocate from tissues (e.g., the lungs, gastrointestinal (GI) tract) when the plasma refill rate is exceeded. RRT reduces both water and osmotically active molecules, thus the efficiency of water removal by RRT mainly depends on transcapillary refill rate [78]. A repaired and intact glycocalyx is required for fluid to remain intravascular [79, 80].

As an adjunct to fluid minimization and active de-resuscitation therapy, the application of leg compression bandages may be considered with the rationale of increasing the interstitial pressure and, therefore, reducing capillary leak, unless contraindications exist. Additionally, lymphatic drainage may be increased. This method was successful in patients with septic shock as well as liver transplant recipients [81, 82], however, firm evidence is still awaited. Importantly, contraindications for leg compressions, such as peripheral arterial disease, a history of peripheral bypass, and local skin or soft tissue conditions should be recognized [83].

Whether fluid de-resuscitation actually leads to improved critical care outcomes is currently uncertain. A recently published meta-analysis on de-resuscitation in patients with septic shock showed no difference in survival with the use of de-resuscitation measures [3]. The signal favours usual care over fluid de-resuscitation in this analysis [3]. However, in this investigation, only three out of five RCTs currently published on active de-resuscitation measures in patients with sepsis/septic shock achieved fluid separation between groups [3]. Silversides and colleagues evaluated in the RADAR-2 trial the feasibility of active fluid removal in the general ICU population and demonstrated a significant fluid separation in the intervention group [84]. However, the trial was not designed to assess patient-centred outcomes. The latter trial combined pharmacological and mechanical (RRT) measures to achieve de-resuscitation [84]. Recently, the POINTCARE-2 study, a stepped wedge cluster open-label randomized controlled trial, and the first published de-resuscitation study which was powered to assess clinical outcomes, revealed that a structured de-resuscitation protocol that combined a weight-driven fluid restriction, diuretics and ultrafiltration did not reduce 60-day mortality [13]. Of the pre-defined safety outcomes, only hypernatremia was more frequent in the structured de-resuscitation group [13]. A further large multi-centre study in the general ICU population investigating early goal-directed therapy with is currently recruiting and has already included more than 50% of the required patients [85]. Further high-quality studies on de-resuscitation measures evaluating patient-centred outcomes are highly warranted.

## When shall I start and stop FAS treatment?

**Fig. 2:**
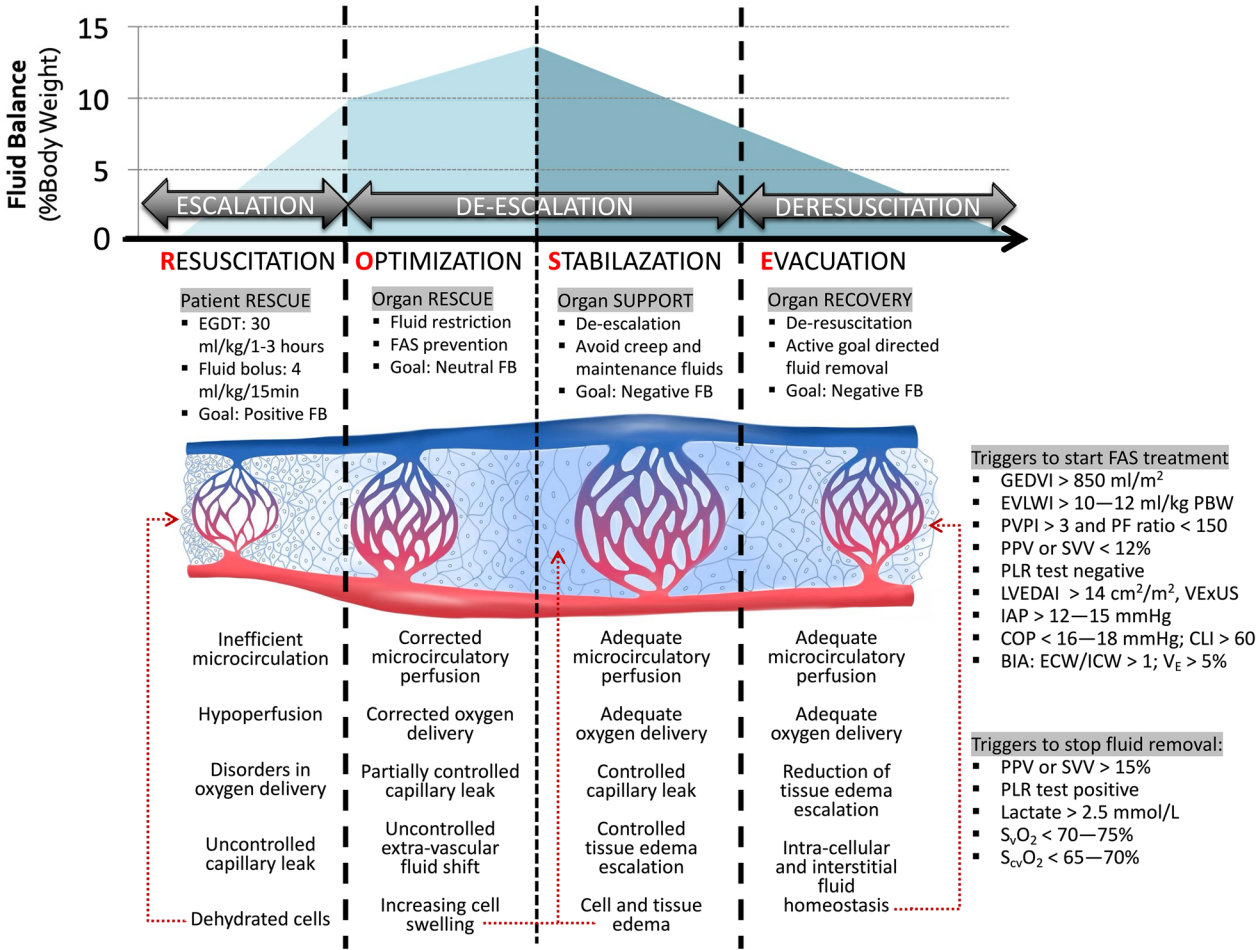
The 4 phases conceptual ROSE model and deleterious effects of fluid accumulation syndrome. Adapted with permission from Malbrain et al. according to the Open Access CC BY Licence 4. 0 [15–17]. Artwork kindly provided by Dr Ricardo Castro, Pontificia Universidad Católica de Chile, Chile. *IAP*: intra-abdominal pressure, *BIA*: bio-impedance analysis, *COP*: colloid oncotic pressure, *ECW/ICW*: extracellular/intracellular water, *EVLWI*: extra-vascular lung water index, *GEDVI*: global end-diastolic volume index, *IVCCI*: inferior vena cava collapsibility index, *LVEDAI*: left ventricular end-diastolic area index, *MAP*: mean arterial pressure, *OCS*: ocular compartment syndrome, *PAOP*: pulmonary artery occlusion pressure. *PLR*: passive leg raising, *PPV*: pulse pressure variation, *PVPI*: pulmonary vascular permeability index, *RVEDVI*: right ventricular end-diastolic volume index, *RVR*: renal vascular resistance, *S*_*cv*_*O*_*2*_: central venous oxygen saturation, *S*_*v*_*O*_*2*_: mixed venous oxygen saturation, *SV*: stroke volume, *SVV*: stroke volume variation

Fluid de-resuscitation should only start when the patient is fluid unresponsive, signs of tissue hypoperfusion are absent, and signs of FAS present. The main concern for fluid removal that is too early or too fast is hypovolemia and the subsequent hemodynamic instability and tissue hypoperfusion. There is currently no gold standard for the safety criteria of fluid de-resuscitation. Some potential criteria for when to start and when to stop FAS treatment are shown in Fig. 2.

## Conclusions

This comprehensive review underlines the importance of FAS in patients with sepsis/septic shock. There is currently a lack of international consensus on diagnosis and monitoring tools of FAS. Prevention of FAS is as important as treatment. Therefore, a differentiated individualized stepwise approach, including minimizing fluid intake (e.g., limiting IV fluids and de-escalation whenever possible) and maximizing fluid output (e.g., by diuretics or combination therapy or renal replacement therapy with net ultrafiltration) depending on the patient’s current phase of septic shock and fluid requirement is needed. Treatment of FAS is symptomatic as there are currently no viable treatment options for the underlying problem of capillary leakage/increased vascular permeability. This huge knowledge gap requires research and evidence for clinicians to be able to therapeutically target the underlying disease and pathophysiology. However, with no such therapeutic targets and targeted inventions available, the only option is symptomatic treatment. Symptomatic treatment is extremely complex and requires a personalized approach as we try to treat the right patient (depending on the underlying disease, i.e. sepsis/septic shock) at the right time (i.e., stages of the ROSE model) with the right intervention (fluids, preventive measures, de-resuscitation measures). Thus, fluid management strategies should not be “liberal” or “restrictive” but patient-centered and individualized.

Future studies should focus on the different triggers, targets and safety limits to initiate and stop de-resuscitation, as well as the potential side effects of de-resuscitation that is inappropriately early, late, too rapid, too long, too little, or too liberal. The impact of FAS and de-resuscitation on capillary leak and the integrity of the endothelial glycocalyx requires further investigation, as does the joint application of vasopressors with fluid therapy (indication, dose, duration, and how to balance with fluid therapy).

## Data Availability

Not applicable.
