# Correction: Fluid accumulation syndrome in sepsis and septic shock: pathophysiology, relevance and treatment—a comprehensive review

**DOI:** 10.1186/s13613-024-01403-1

**Published:** 2025-01-31

**Authors:** Carmen Andrea Pfortmueller, Wojciech Dabrowski, Rob Wise, Niels van Regenmortel, Manu L. N. G. Malbrain

**Affiliations:** 1 Department of Intensive Care, Inselspital, Bern University Hospital and University of Bern, Freiburgstrasse 10, 3010 Bern, Switzerland; 2 First Department of Anaesthesiology and Intensive Therapy, Medical University of Lublin, Lublin, Poland; 3 Department of Anaesthesia and Critical Care, School of Clinical Medicine, University of KwaZulu-Natal, Durban, South Africa; 4 Faculty Medicine and Pharmacy, Vrije Universiteit Brussel (VUB), Brussels, Belgium; 5 Intensive Care Department, John Radcliffe Hospital, Oxford University Trust Hospitals, Oxford, UK; 6 Department of Intensive Care Medicine, Ziekenhuis Netwerk Antwerpen Campus Stuivenberg/Cadix, Antwerp, Belgium; 7 Department of Intensive Care Medicine, Antwerp University Hospital, Antwerp, Belgium; 8 International Fluid Academy, Lovenjoel, Belgium; 9 Medical Data Management, Medaman, Geel, Belgium

**Correction: Annals of Intensive Care (2024) 14:115** 10.1186/s13613-024-01336-9

Following publication of the original article, the authors removed the word “prevention” in Table 1 as it is already in the table title and the title of Table 2 has been corrected. Table 3 has been edited. The corrected tables 1, 2 and 3 are given below.

Incorrect Table 1

**Table 1:** Prevention and monitoring of fluid accumulation syndrome (FAS)

Prevention	NOTE: Fluids should only be administrated when hypovolemia, fluid responsiveness AND signs of impaired tissue perfusion are present
	*Monitoring* – Basic monitoring (i.e. arterial and central venous line), – in case of unresolved shock consider echocardiography and advanced hemodynamic monitoring (eg. pulse contour or transpulmonary thermodilution) – obtain baseline body weight (scale, estimate, retrieve from medical records) – Monitor for risk for fluid accumulation: i.e. daily body weight, daily and cumulative fluid balance, edema formation, sonography – Assess for impaired end-organ function: IAP, APP, PF ratio, success of EN, EVLWI, PVPI, BIA – Assess fluid responsiveness with functional hemodynamics (i.e. PPV or SVV, passive leg raising test, end-expiratory occlusion test) – Assess for signs of tissue hypoperfusion (DO_2_/VO_2_ mismatch; i.e. elevated lactate, increased mottling score, increased capillary refill time)
	*Prevention* A) Fluids are required – use a restrictive fluid management regime – when maintenance fluids are necessary, opt for balanced and sodium-poor alternatives (NaCl 0.18–0.45%) – Use low-chloride alternatives to NaCl 0.9% when selecting resuscitation and replacement fluids – frequently re-assess preload and fluid responsiveness/tissue perfusion, only administer fluids in fluid responsive patients – consider early norepinephrine use – stop fluid administration once fluid responsiveness and/or tissue perfusion are absent – consider the (early) use of albumin 20%, especially when serum albumin levels are low (< 30g/L) [1] B) Fluids are not required: de-escalation – Limit fluid intake – Limit sodium intake – Limit/avoid maintenance solutions – Limit/avoid fluid creep – Improve lymphatic drainage (i.e. use leg compression bandages) – Use high density or concentrated enteral formula’s (i.e. 2 kcal/ml)

Table adapted with permission from Malbrain et al. according to the Open Access CC BY Licence 4.0 (ESM file) [24]

This table presents some suggestions for prevention of fluid accumulation based on personal experience of the co-authors. It does not aim to provide an exhaustive, graded and concise overview of the literature as current evidence is mostly limited to observational, retrospective or small clinical studies, and more randomized trials are needed to better establish a personalized approach to fluid management. For more information we refer the reader to some recent review papers on this topic [23, 92]

*EN*: enteral nutrition, *EVLWI*: extra-vascular lung water index, *FA*: fluid accumulation, *APP*: abdominal perfusion pressure (MAP minus IAP), *IAP*: intra-abdominal pressure, *PEEP*: positive end-expiratory pressure, *PF*: P_a_O_2_ over F_i_O_2_ ratio, *PPV*: pulse pressure variation, *CVVH*: continuous veno-venous hemofiltration, *IAP*: intra-abdominal pressure, *MAP*: mean arterial pressure, *PPV*: pulse pressure variation, *RRT*: renal replacement therapy, *SVV*: stroke volume variation, *UF*: ultrafiltration, *BIA*: bio-electrical impedance analysis

Correct Table 1

**Table 1:** Prevention and monitoring of fluid accumulation syndrome (FAS)

NOTE: Fluids should only be administrated when hypovolemia, fluid responsiveness AND signs of impaired tissue perfusion are present
*Monitoring* – Basic monitoring (i.e. arterial and central venous line), – in case of unresolved shock consider echocardiography and advanced hemodynamic monitoring (eg. pulse contour or transpulmonary thermodilution) – obtain baseline body weight (scale, estimate, retrieve from medical records) – Monitor for risk for fluid accumulation: i.e. daily body weight, daily and cumulative fluid balance, edema formation, sonography – Assess for impaired end-organ function: IAP, APP, PF ratio, success of EN, EVLWI, PVPI, BIA – Assess fluid responsiveness with functional hemodynamics (i.e. PPV or SVV, passive leg raising test, end-expiratory occlusion test) – Assess for signs of tissue hypoperfusion (DO_2_/VO_2_ mismatch; i.e. elevated lactate, increased mottling score, increased capillary refill time)
*Prevention* A) Fluids are required – use a restrictive fluid management regime – when maintenance fluids are necessary, opt for balanced and sodium-poor alternatives (NaCl 0.18–0.45%) – Use low-chloride alternatives to NaCl 0.9% when selecting resuscitation and replacement fluids – frequently re-assess preload and fluid responsiveness/tissue perfusion, only administer fluids in fluid responsive patients – consider early norepinephrine use – stop fluid administration once fluid responsiveness and/or tissue perfusion are absent – consider the (early) use of albumin 20%, especially when serum albumin levels are low (< 30g/L) [1] B) Fluids are not required: de-escalation – Limit fluid intake – Limit sodium intake – Limit/avoid maintenance solutions – Limit/avoid fluid creep – Improve lymphatic drainage (i.e. use leg compression bandages) – Use high density or concentrated enteral formula’s (i.e. 2 kcal/ml)

Table adapted with permission from Malbrain et al. according to the Open Access CC BY Licence 4.0 (ESM file) [2]

This table presents some suggestions for prevention of fluid accumulation based on personal experience of the co-authors. It does not aim to provide an exhaustive, graded and concise overview of the literature as current evidence is mostly limited to observational, retrospective or small clinical studies, and more randomized trials are needed to better establish a personalized approach to fluid management. For more information we refer the reader to some recent review papers on this topic [3, 4]

*EN*: enteral nutrition, *EVLWI*: extra-vascular lung water index, *FA*: fluid accumulation, *APP*: abdominal perfusion pressure (MAP minus IAP), *IAP*: intra-abdominal pressure, *PEEP*: positive end-expiratory pressure, *PF*: P_a_O_2_ over F_i_O_2_ ratio, *PPV*: pulse pressure variation, *CVVH*: continuous veno-venous hemofiltration, *IAP*: intra-abdominal pressure, *MAP*: mean arterial pressure, *PPV*: pulse pressure variation, *RRT*: renal replacement therapy, *SVV*: stroke volume variation, *UF*: ultrafiltration, *BIA*: bio-electrical impedance analysis

Incorrect Table 2

**Table 2:** Terminology

Resuscitation fluids	Resuscitation fluids refer to the fluids administrated in the early initial phase of shock to restore of adequate organ perfusion. They should only be given in case of shock (DO_2_/VO_2_ imbalance with increased lactate) AND low preload AND fluid responsiveness and they should always be given as a fluid challenge i.e. assessing fluid status and fluid responsiveness before and after. Most often they are given as a bolus of 4ml/kg over 10–15 min [93]
Fluid Creep	A term that refers to the unintentional and unmeasured fluid volumes administered in the process of delivering other medication (antibiotics, sedatives, painkillers, etc.) and/or nutrition through enteral and parenteral routes
Maintenance fluids	Maintenance fluids are a source of water, electrolytes and also potentially glucose. The aim of maintenance fluid is to cover the daily needs and prevent dehydration and electrolyte disorders, if the patients need is not met through other sources (i.e. nutrition)
De-escalation	Refers to not initiating extra fluids (withhold) or lowering of the dose or speed of administration (withdraw/reduction) of previously started fluid therapy due to improvement in the clinical condition of the patient
De-resuscitation	Correction of fluid accumulation or fluid overload by active removal of the excess fluids using non-pharmacological mechanical (e.g., dialysis with net ultrafiltration) or pharmacological (e.g., diuretics) methods

Fluid creep may sum up to 33% of all fluids administered, compared to maintenance/replacement (25%), nutrition (33%) and resuscitation (6%) [55]

Correct Table 2

**Table 2:** Terminology

Resuscitation fluids	Resuscitation fluids refer to the fluids administrated in the early initial phase of shock to restore of adequate organ perfusion. They should only be given in case of shock (DO_2_/VO_2_ imbalance with increased lactate) AND low preload AND fluid responsiveness and they should always be given as a fluid challenge i.e. assessing fluid status and fluid responsiveness before and after. Most often they are given as a bolus of 4ml/kg over 10–15 min [5]
Fluid Creep	A term that refers to the unintentional and unmeasured fluid volumes administered in the process of delivering other medication (antibiotics, sedatives, painkillers, etc.) and/or nutrition through enteral and parenteral routes
Maintenance fluids	Maintenance fluids are a source of water, electrolytes and also potentially glucose. The aim of maintenance fluid is to cover the daily needs and prevent dehydration and electrolyte disorders, if the patients need is not met through other sources (i.e. nutrition)
De-escalation	Refers to not initiating extra fluids (withhold) or lowering of the dose or speed of administration (withdraw/reduction) of previously started fluid therapy due to improvement in the clinical condition of the patient
De-resuscitation	Correction of fluid accumulation or fluid overload by active removal of the excess fluids using non-pharmacological mechanical (e.g., dialysis with net ultrafiltration) or pharmacological (e.g., diuretics) methods

Fluid creep may sum up to 33% of all fluids administered, compared to maintenance/replacement (25%), nutrition (33%) and resuscitation (6%) [6]

Incorrect Table 3

**Table 3:**
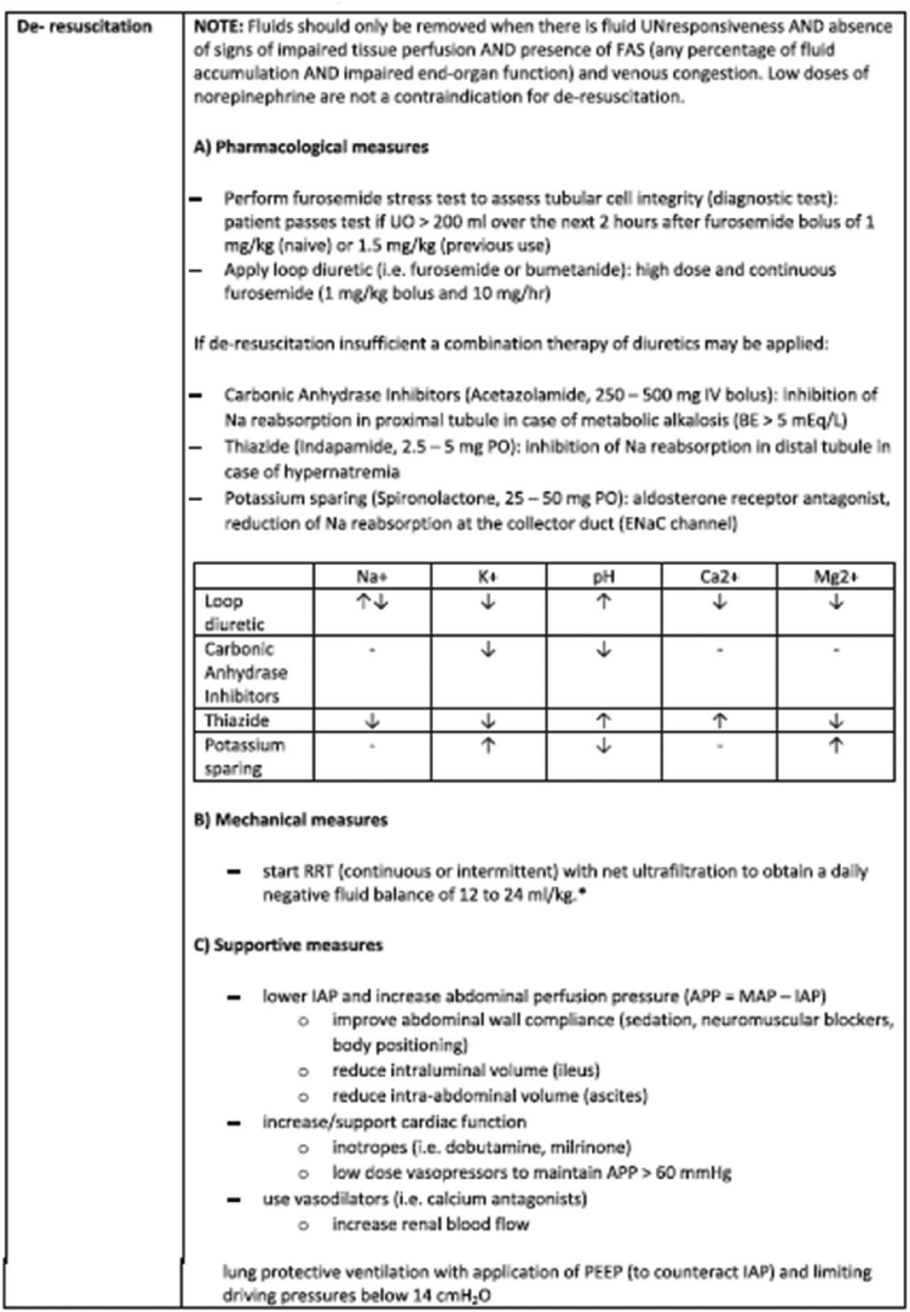
Treatment of fluid accumulation syndrome (FAS)

Correct Table 3

**Table 3:**
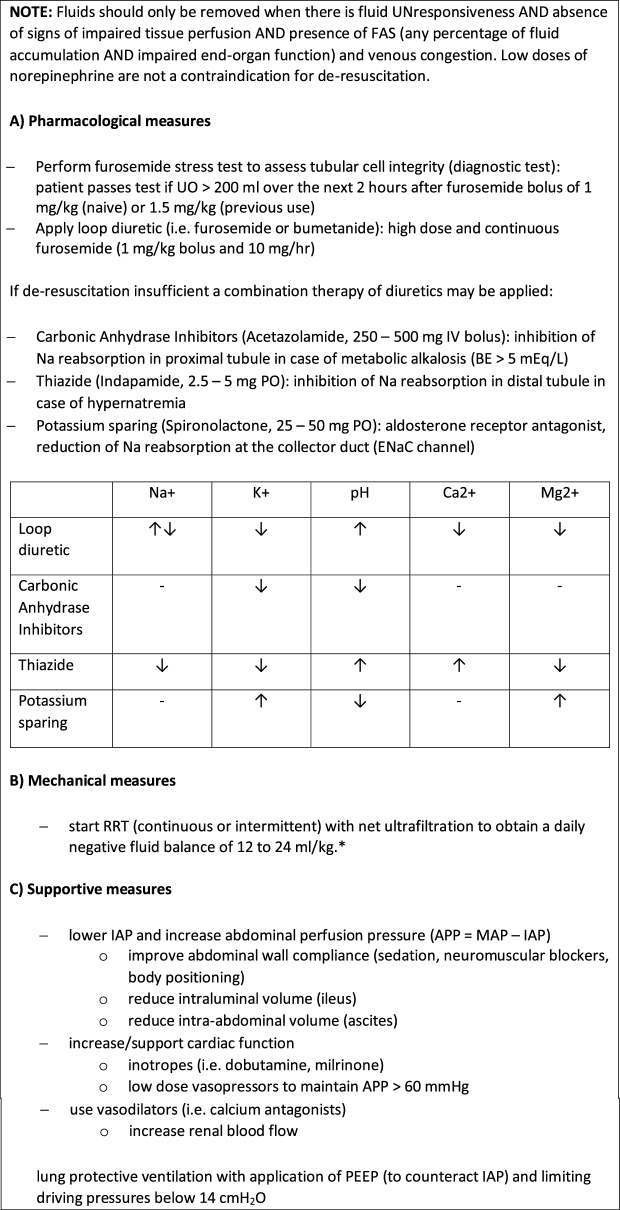
Treatment (de-resuscitation) of fluid accumulation syndrome (FAS)

Table adapted with permission from Malbrain et al. according to the Open Access CC BY Licence 4.0 (ESM file) [1]

This table presents some suggestions for prevention and treatment of fluid accumulation based on personal experience of the co-authors. It does not aim to provide an exhaustive, graded and concise overview of the literature as current evidence is mostly limited to observational, retrospective or small clinical studies, and more randomized trials are needed to better establish a personalized approach to fluid management. For more information we refer the reader to some recent review papers on this topic (11, 12).

*EN*: enteral nutrition, *EVLWI*: extra-vascular lung water index, *FA*: fluid accumulation, *APP*: abdominal perfusion pressure (MAP minus IAP), *IAP*: intra-abdominal pressure, *PEEP*: positive end-expiratory pressure, *PF*: P_a_O_2_ over F_i_O_2_ ratio, *PPV*: pulse pressure variation, *CVVH*: continuous veno-venous hemofiltration, *IAP*: intra-abdominal pressure, *MAP*: mean arterial pressure, *PPV*: pulse pressure variation, *RRT*: renal replacement therapy, *SVV*: stroke volume variation, *UF*: ultrafiltration, *BIA*: bio-electrical impedance analysis

*Target net ultrafiltration needs to be tailored to the individual patient and adjusted based on the haemodynamics and fluid requirements of the patient. It may need to be slower in patients with cardiogenic shock and higher in patients who are very fluid overloaded and also have large fluid requirements, ie blood products, TPN etc.

The authors identified an error in Figs. 1, 2 and 3. Figure 1 has now been replaced by a Textbox 1 on the critical appraisal of the definition for fluid accumulation syndrome. Figure 2 then became Figure 1 and Figure 3 became a modified Figure 2. The corrected figures are given below as well as the textbox. 

Incorrect Fig. 1

**Fig. 1:**
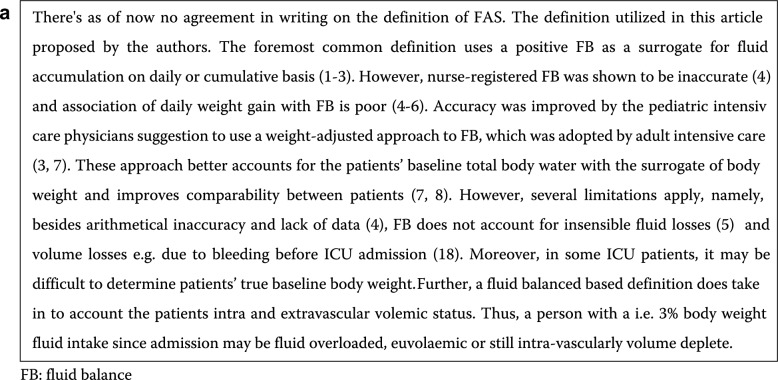
Critical appraisal of fluid accumulation syndrome (FAS)

Correct Fig. 1

**Fig. 1:**
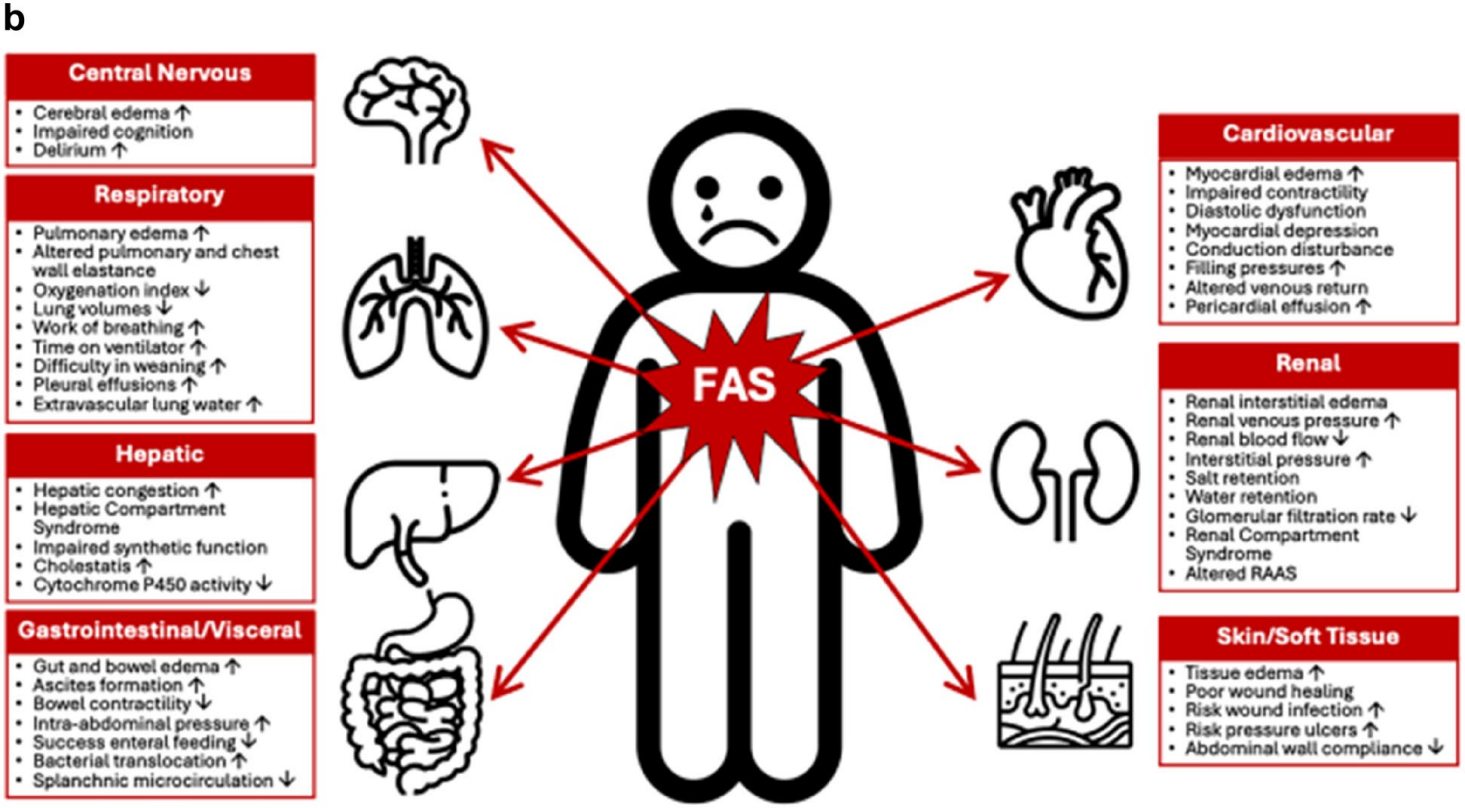
Potential adverse consequences of fluid accumulation. Adapted with permission from Malbrain et al. according to the Open Access CC BY Licence 4.0 [3, 7, 8]. Effects mentioned are related to the setting of sepsis, capillary leak and fluid accumulation. I.e. the numbness refers to the presence of peripheral edema and anasarca that may cause skin conduction disturbances, compression of nerves, reduced blood flow and reduced mobility. Additionally, severe and prolonged fluid imbalances can lead to a range of health issues and complications, including electrolyte imbalances, which may indirectly affect the body's ability to respond to stress, including the production of cortisol by the adrenal glands. *APP* abdominal perfusion pressure (MAP minus IAP), *RSB* rapid shallow breathing, *HCS* hepatic congestion, *GRV* gastro-esophageal reflux, *CARS* cardiac-renal syndrome, *AKI* acute kidney injury, *JVP* jugular venous pressure, *HJR* hepato-jugular reflux

Incorrect Fig. 2

**Fig. 2:**
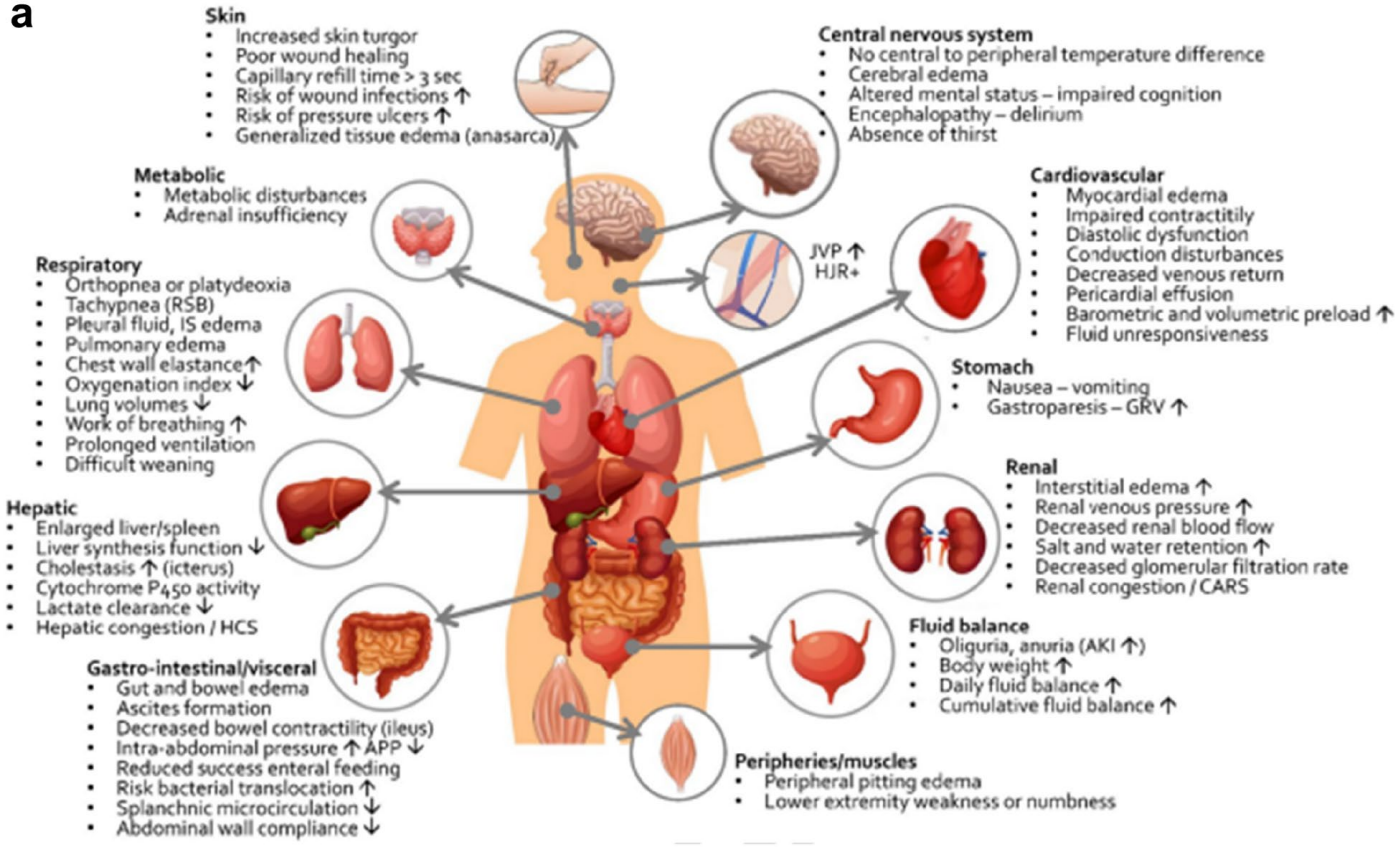
Potential adverse consequences of fluid accumulation. Adapted with permission from Malbrain et al. according to the Open Access CC BY Licence 4.0 [3, 7, 8]. Effects mentioned are related to the setting of sepsis, capillary leak and fluid accumulation. I.e. the numbness refers to the presence of peripheral edema and anasarca that may cause skin conduction disturbances, compression of nerves, reduced blood flow and reduced mobility. Additionally, severe and prolonged fluid imbalances can lead to a range of health issues and complications, including electrolyte imbalances, which may indirectly affect the body's ability to respond to stress, including the production of cortisol by the adrenal glands. *APP* abdominal perfusion pressure (MAP minus IAP), *RSB* rapid shallow breathing, *HCS* hepatic congestion, *GRV* gastro-esophageal reflux, *CARS* cardiac-renal syndrome, *AKI* acute kidney injury, *JVP* jugular venous pressure, *HJR* hepato-jugular reflux

Correct Fig. 2

**Fig. 2:**
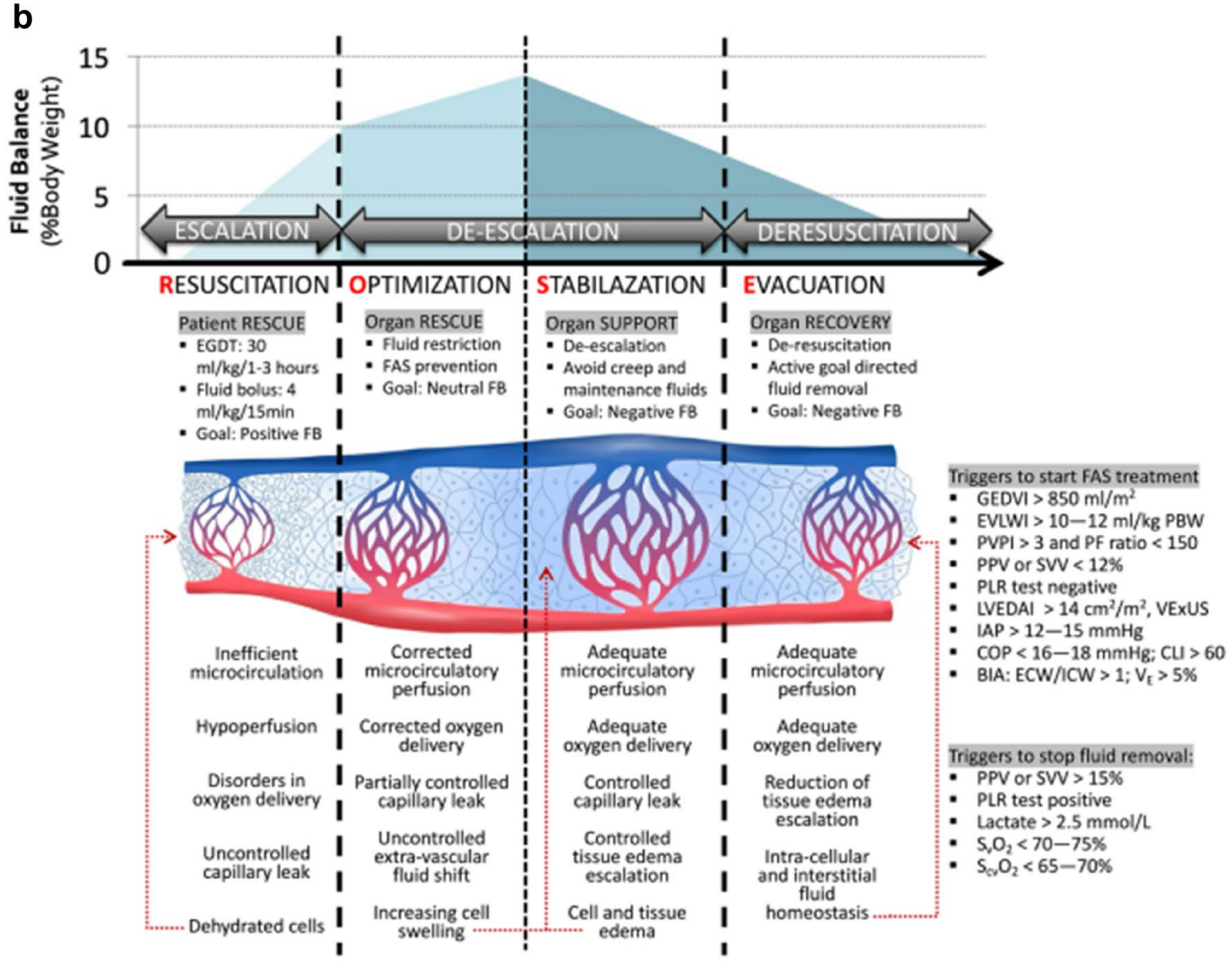
The 4 phases conceptual ROSE model and deleterious effects of fluid accumulation syndrome**.** Adapted with permission from Malbrain et al. according to the Open Access CC BY Licence 4.0 [3, 7, 8]. *IAP:* intra-abdominal pressure*, BIA:* bio-impedance analysis, *COP* colloid oncotic pressure, *ECW/ICW* extracellular/intracellular water, *EGDT* early goal directed therapy, *EVLWI* extra-vascular lung water index, *FAS* fluid accumulation syndrome*, FB* fluid balance*, GEDVI* global end-diastolic volume index, *IVCCI* inferior vena cava collapsibility index, *LVEDAI* left ventricular end-diastolic area index, *MAP* mean arterial pressure, *OCS* ocular compartment syndrome, *PAOP* pulmonary artery occlusion pressure, *PLR* passive leg raising, *PPV* pulse pressure variation, *PVPI* pulmonary vascular permeability index, *RVEDVI* right ventricular end-diastolic volume index, *RVR* renal vascular resistance, *S*_*cv*_*O*_*2*_ central venous oxygen saturation, *S*_*v*_*O*_*2*_ mixed venous oxygen saturation, *SV* stroke volume, *SVV* stroke volume variation. Artwork kindly provided by Dr Ricardo Castro, Pontificia Universidad Católica de Chile, Chile

The original article has been corrected.
